# NMR-Based Chromatography Readouts: Indispensable Tools to “Translate” Analytical Features into Molecular Structures

**DOI:** 10.3390/cells11213526

**Published:** 2022-11-07

**Authors:** Christoph Seger, Sonja Sturm

**Affiliations:** 1 Institute of Pharmacy/Pharmacognosy, CCB—Centrum of Chemistry and Biomedicine, University of Innsbruck, Innrain 80-82, A-6020 Innsbruck, Austria; 2 Labordiagnostic St. Gallen West AG, Zürcherstrasse 505, CH-9015 St. Gallen, Switzerland

**Keywords:** HPLC, HPLC-NMR, HPLC-SPE-NMR, capNMR, NMR spectroscopy, structure elucidation, mass spectrometry, liquid chromatography, HPLC–MS/MS, metabolomics

## Abstract

Gaining structural information is a must to allow the unequivocal structural characterization of analytes from natural sources. In liquid state, NMR spectroscopy is almost the only possible alternative to HPLC-MS and hyphenating the effluent of an analyte separation device to the probe head of an NMR spectrometer has therefore been pursued for more than three decades. The purpose of this review article was to demonstrate that, while it is possible to use mass spectrometry and similar methods to differentiate, group, and often assign the differentiating variables to entities that can be recognized as single molecules, the structural characterization of these putative biomarkers usually requires the use of NMR spectroscopy.

## 1. Introduction

### 1.1. The Need for Structure Elucidation

In deductive science, especially when heuristic approaches are or must be chosen, it is to be expected that an analytical development of an object of investigation offers the researcher more questions than answers. It is up to the design of the research approach whether this circumstance is understood as an invitation for further investigations or whether open questions are accepted, discussed, and filed as inconclusive results. Gaining knowledge about processes by collecting qualitative and quantitative data is an essential methodological approach in the natural sciences. Analytical measurements, which aim to collect observational data in a controlled, rational, and reproducible manner, link individual study objects with hypotheses to be tested [1]. Such experiments can truly be described as ubiquitous. Their application spans all fields of science—from particle physics to continent-spanning ecological projects with dozens of scientists collecting highly diverse datasets. Even in modern (human) medicine, classical empirical approaches are increasingly being replaced by "evidence-based medicine", a hypothesis falsification approach relying on statistical data analysis and an almost dogmatic belief in the measurability of treatment effects [2].

Modern natural science has several spectroscopic or spectrometric methods at its disposal that have been developed in recent decades and are that continue to evolve [3,4]. Whenever molecules need to be unequivocally detected, identified, and quantified, instrumental analysis has become an indispensable tool. Methodologically, it can be divided into two areas—analyte separation and analyte identification.

In analyte separation, the chemical entities under investigation are separated from the complex, ubiquitous and often interfering sample matrix background. To identify the analytes, the physical, biological, or chemical properties of the molecules under investigation are measured. Analyte identification is not necessarily preceded by analyte separation, especially when highly analyte-specific detection systems are available. In many cases, analytical instruments used to detect an analyte can also provide quantitative data, meaning that a reproducible correlation of a signal expression (most often the signal strength) and the analyte quantity can be demonstrated. Taken together, the combination of high-performance separation systems with highly selective analyte detection techniques is widely recognized as a successful strategy that meets the needs of modern science.

At this point, it must clearly be stated that not all research approaches require a clear assignment of the obtained measurement signals to individual molecules and thus to identifiable analytes. It is also common knowledge that whether the signals obtained can be translated into structural information is a question of the structural resolution capability of the analysis methods employed. Therefore, the successful use of spectroscopic measurements does not necessarily lead to data that enable the knowledgeable researcher to clearly identify individual molecular species, and it is often not needed to solve the scientific questions under investigation.

For example, in NIR spectroscopy which, due to its experimental properties, occupies a prominent position in the investigation of complex matrices, the observed vibrational resonances are so complex that even the identification of substance classes is only possible to a limited extent [5]. Nonetheless, the method can be used to differentiate sample sets through its combined use with interpretive chemometric methods such as principal component analysis (PCA) or discriminant analysis (DA) [6]. The identified principal components (PCs), which are correlated with spectral ranges in the sense of a complex linear combination, are “super variables” made up of a weighted linear combination of an unknown number of unidentified molecules. PCs have the status of surrogate parameters (dataset “features”) allowing the differentiation of the investigated samples with the correlation strength to discriminate spectral regions sets known as “loadings”. Whenever the identity of these molecules is an objective in the research question, or further questions arise during the interpretation of the PC-derived results, which must be pursued as new objectives, it is necessary to choose alternative investigation approaches allowing the unequivocal identification of the putative “biomarkers”.

This statement is of course not limited to NIR spectroscopy. It also applies to many other spectroscopic and spectrometric technologies used in life science. If the interpretation of the data generated by the applied method does not allow conclusions about the underlying molecules, the use of reference substances or comparison with relevant databases is required to allow at least a preliminary (tentative) signal/analyte correlation. Whilst UV/VIS and IR spectroscopy usually only allow the identification of one substance class, since the signals/signal groups are always generated only by molecule parts (chromophores, functional groups), the more holistic methods such as mass spectrometry (MS) and NMR spectroscopy can be used to completely characterize molecules in terms of the uniform detection of all molecule parts. However, it is perfectly clear and known to the reader that the fundamental difference between MS and NMR spectroscopy is that the signals of MS are the *m/z* ratios of ions generated from the molecules in the gas phase, whereas in NMR spectroscopy, atomic properties intrinsic to the molecule (the nuclear spin) are observed.

### 1.2. Mass Spectrometry-Based Analysis Readout

The signals of the ions of a molecule detected in mass spectrometry are the result of chemical and physical gas-phase reactions in which the molecule undergoes a transformation (destruction). The relative intensities of the ions that occur are a function of the kinetic and thermodynamic reaction conditions. The ion ratios are therefore extremely dependent on both the molecular properties and the experimental conditions including the velocity and density of the molecules, the collision probabilities, and the activation energies of the reactions. The interpretation of MS signals, e.g., the correlation with the structural features of the investigated molecules or the derivation of a molecular formula, strongly depends on the instrumental design of the mass spectrometer used.

In gas chromatography–mass spectrometry (GC–MS), the standardized 70 eV EI source delivers mass spectra that are so well reproducible that very often good (tentative) substance identifications are possible through comparison with databases. It is also well perceived that only when using high-resolution mass spectrometers can the detected ions be correlated with molecular formulae [7]. The low-resolution mass selectors used, for example, in “triple quadrupole” (QqQ) type tandem mass spectrometers for quantitative analysis, typically result in feature-poor mass spectra that are so poorly reproducible between different types of mass spectrometers that they have very limited use for analyte characterization unless databases are employed [8].

Consequently, if one wants to work on complex questions in the life sciences with mass spectrometric technologies, one is basically always faced with the dilemma that there is hardly any way to translate spectroscopic features into molecular signatures. This is of course also true if one couples the spectrometric domain (MS) with a separating technology (e.g., high-performance liquid chromatography (HPLC), gas chromatography (GC), or capillary electrophoresis (CE)), as this serves to simplify the analytical problem in the mass spectrometric domain by separating the analytes of interest as much as possible before ion generation [9,10].

Applications where the analysis of data from HPLC-MS couplings, i.e., the classical bioanalytically used mass spectrometric data, provide sufficient structural information to solve analytical problems in terms of unambiguous analyte identification, and are found in both macromolecule and small molecule research. In the case of linearly organized bio-macromolecules with a limited number of building blocks (e.g., peptides, proteins, and DNA/RNA oligomers), it is possible to analyze the fragmentation of the molecules in the mass spectrometer in such a way that the sequential arrangement of the building block elements can be deduced. These approaches are one of the most important basic scientific elements of modern proteome and genome analysis. Whenever the number of observable elements can be restricted, i.e., when the research questions move from an undirected analysis of biological diversity to focusing on a partial aspect, the database-oriented identification can be helpful. This approach is used in toxicology and forensics when a “general unknown” screening is performed, but it is also of importance when the structurally less diverse primary elements of metabolism are qualitatively and quantitatively investigated.

In research approaches attempting to interpret the most holistic data possible from biological matrices (“untargeted metabolomics approaches”), the spectral signature is collected in a “feature matrix” (for HPLC-MS, e.g., retention times, masses of the ions, etc.) together with the differentiating metadata of the samples after a few steps of data post-processing and fed into postanalytical multivariate data processing (“data mining”) algorithms establishing multi-dimensional correlation networks [11,12,13,14].

### 1.3. NMR Spectroscopy-Based Analysis Readout

If, on the other hand, NMR spectroscopy is used as the technological basis of a holistic metabolomics technology, the analytical evidence is quite different. Signals in an NMR molecular spectrum do not arise from a chemical reaction of the molecule but are an inherent property of the same. NMR signals are signals from individual atoms of the molecule and depend on both the observed nuclear species and the molecular environment of the observed atom (“chemical shift”). The signal intensities are directly proportional to the relative number of observed nuclei, and the neighboring nuclei in scalar networks are correlated by the effect of quantum coherence (“scalar coupling”) [15].

In liquid phase samples, nuclear magnetic resonance (NMR) spectroscopy has a long standing as analytical technique. In recent decades, it has primarily been utilized as a structure elucidation or structure confirmation tool, where it has its unquestioned advantages over other spectroscopic methods including mass spectrometry, but can also be used for analyte quantification from mixtures [16]. It is considered one of the major cornerstones for establishing molecular structures by analyzing connectivity networks at the atomic level. In contrast to mass spectrometry, which has accompanied structure elucidation efforts in natural product research as a standalone technique for more than half a century and having become an easily applicable HPLC detector by the maturation of John B. Fenn’s invention of the atmospheric pressure ionization (API) technique ESI (electrospray ionization) in recent decades, NMR spectroscopy is still not a widespread detector device for liquid chromatography setups.

### 1.4. NMR Data Quality and Structure Elucidation

It shall not be ignored that, even after forty years of development in nuclear magnetic resonance (NMR) spectroscopy, total synthesis is still the final proof for a structure proposal. This holds especially true if complex ring systems or a demanding alkaloid are encountered [17]. To address the problem of ambiguity in NMR data interpretation, to reduce the enormous loss of information due to the interpretation of chemical shifts, correlation signals, and coupling constants, and to reduce the loss of NMR data due to only local data storage, the NMR raw data initiative aims to advance the global standardization of NMR data storage and NMR data interpretation [18].

The possibility of performing ab initio calculations of NMR signal positions (chemical shifts, couplings) of chemical substances based on the traceability of NMR measurements to quantum mechanical principles allows to verify structure hypotheses based on proton NMR experimental data via HiFSA [19,20]. If ^13^C-NMR spectroscopy is utilized, e.g., in the CSEARCH or the ACD approach, the knowledge of the dependence of the chemical shift on the electron density at the observed nucleus allows to confirm or falsify the assignments of NMR signals to specific atomic positions in molecules [21]. Computer-assisted structure elucidation (CASE) approaches utilizing 1D and 2D-NMR data might develop valuable tools to assist the researcher striving to perform the de novo structure characterization of analytes [22,23]. Combining NMR signal processing with algorithms allowing NMR spectra prediction and aiding structure verification are of exceptional value for researchers, if integrated in available software packages [24]. Finally, the availability of generated NMR data in publicly accessible databases has the potential to minimize the amount of work involved in structure elucidation by spectra comparison. This depends, of course, on the deposited spectra and the assigned structures being checked for reproducibility and validity. Both the present and especially the historical data situations allow one to be quite skeptical in this respect [25,26], but initiatives such as NMReDATA might solve this problem in the near future [27,28].

Taken together, gaining additional structural information is a must to allow the unequivocal structural characterization of analytes from natural sources. The purpose of this review article was to demonstrate that while it is possible to use mass spectrometry and similar methods to differentiate, group, and often assign the differentiating variables to entities that can be recognized as single molecules, the structural characterization of these putative biomarkers usually requires the use of NMR spectroscopy. This overview is based on three previous publications by the authors [10,15,16]. Based on the findings reported therein, some of which are repeated in Section 3, Section 4 and Section 5 of this review, the readership is informed about the latest developments in this field. The aim was to awaken the understanding that the structural elucidation of organic molecules as a central building block in gaining scientific knowledge in the dereplication of the diversity of natural products must not be disregarded.

## 2. Molecule-Centered Plant Biochemistry and Phytochemistry

### 2.1. Need of Structure Elucidation in Plant Biochemistry and Phytochemistry

Classical phytochemical work has been characterized for decades by the isolation and unequivocal identification of individual natural substances to link bioactivities associated with organisms to single molecules in mechanistic models [29]. In this context, as explained in the first chapter, structure elucidation is very well understood, NMR technologies are mature, and the knowledge base is broad (see above). At the turn of the century, the concept of “metabolomics”/“metabonomics” [30,31] found its way into phytochemistry [32,33]. With this approach, which was initially intended as an untargeted one, the structural characterization of possible marker features has taken on a new significance, both in terms of its importance for applied research and the complexity of the associated technological tasks [9].

Research approaches that address the metabolic diversity and performance of plants encounter complex patterns of secondary metabolites. In contrast to "primary metabolites," these compounds are produced by adaptive complex metabolic pathways that are biogenetically distinct from the conserved and universal primary mechanisms for maintaining life functions (e.g., photosynthesis, glycolysis, DNA replication). Among the most important secondary classes are terpenoids, alkaloids, polyphenols, and polyketides [34]. A high structural diversity is found within these categories and even structural hybrids combining unrelated biogenetical pathways are often encountered. There are estimated to be hundreds of thousands of secondary metabolites whose spectroscopic data are known. Several lists and databases are available, and the LOTUS initiative aims to provide for the open knowledge management of these sources [35]. If analytical techniques using mass spectrometric detectors (e.g., GC–MS, HPLC–MS, CE–MS) are applied to dereplicate mixtures of natural products, the information density of the mass spectra, which is very limited with respect to the structural identity of natural products, has proven to be a major methodological bottleneck. Hence, in many publications, analytes showing up as discriminators in a specific sample set (the putative biomarkers) have been left unidentified.

Almost two decades ago, Wolfram Weckwerth did conclude his review on metabolomics in system biology with the critical statement that only a small fraction of compounds present are detected in HPLC–MS-based metabolomics investigations, and that from this limited number of molecules, most remain unidentified [36]. He and others reiterated this observation some years thereafter by stating that “a major drawback of metabolomic technologies yet to be overcome is the vast number of unknown compound structures” [37] and that “increasing the number of metabolite identifications within existing profiling platforms is prerequisite for a substantial improved scope of profiling studies” [38].

Analytical research in the 21st century has not only seen immense technological advances, which have also been useful to metabolomics research approaches, but have also been marked by a revolutionary change in data processing in systems biology research approaches. Under the term “Molecular Networking”, a research idea was developed about a decade ago, in which the joint statistical analysis of genomic and phenomic data was performed [39,40].

This approach has also found its way into phytochemical research. First and foremost, the expected technical problems of the analytical platforms were addressed. For example, there is the quite complex question of which chromatographic peaks correspond to a feature, i.e., a molecular entity, despite differences in retention times (based on experimental limitations). Moreover, there is the equally complex task of unambiguously determining which of the mass signals found in the retention region of the hypothesized features that can be associated with it are meaningful for the subsequent work of identification and which merely originate from adducts with components of the matrix [41,42,43,44,45]. In addition, several projects and consortia have attempted to correlate the features identified in the datasets. Signal correlations were measured within an analytical dataset or across datasets from distinctively different analytical sources. One dataset was used, for example, in the reconstruction of possible biomarkers by NMR-STOCSY [46] or in the correlation analysis of ions and retention times in HPLC–MS couplings [47]. An important step was taken when it became possible to combine data from mass spectrometric measurements with NMR data, e.g., via the hetero-covariance method (HetCA) [48] or in molecular networking approaches [49]. If this dataset is evaluated discriminately against the bioactivity data of the individual samples, this interesting approach can be used to combine occurring bioactivities with spectral information in such a way that the identification of the associated molecules can be ensured [50].

From the extensive literature of past years [14], only one should be discussed: in a very extensive study on the secondary metabolite profile of an endophytic marine fungus (*Fusarium petroliphilum*) and the bioactivity against *Staphylococcus aureus* (inhibition of quorum sensing), an attempt was made to dereplicate using all the mentioned techniques [51]. The authors claimed to have clearly identified 22 components, and a further 27 components were structurally characterized by the statistical comparisons of mass spectrometric features with isolated components. This undoubted success in dereplicating the secondary metabolite profile of a difficult-to-access marine organism must, however, be set against the workload and the self-imposed limitations in the selection of the characterized features. From the HPLC-MS spectra, approximately 4000 features were derived, among which approximately 1500 molecular nodes were worked out by statistical correlation techniques. The comparison of these nodes against the GNPS database resulted in approximately 400 (10% of all features, 50% of all nodes) hits. All database results that were not from the phylogenetic relationship of the object under investigation were not considered, which is a strong indication of an inductive guidance of the research project. The researchers imposed a further restriction on themselves by declaring the most intense peaks in the mass spectra to be the most relevant peaks. They concluded their remarks on the identification of secondary metabolites from the study object with the words “as shown […], more than 30% of the most intense MS peaks could not be annotated through this process, which could be either unknown compounds or compounds never reported in the *Nectriaceae* family”. In summary, this shows that even when excellent research infrastructures are used, the chosen statistical approach via molecular networks does not include most features found in the untargeted metabolomics data collection in the identification process. In turn, the identification of metabolites largely depends on the quality of the database solutions (here GNPS), which are generated from the classical phytochemical work. It follows that the untargeted metabolomics approach in this research setting is compared with targeted secondary metabolite data from the past. Any molecular traits that are considered unknown for the group of organisms are intentionally excluded from further analysis. Nonetheless, approximately a dozen natural products were found that have not yet been described, and approximately 50 molecules were characterized. It is not necessary to mention that the characterization of the novel substances required the intensive use of 1D- and 2D-NMR spectroscopy, i.e., reverting to the methods of classical phytochemical structure elucidation.

### 2.2. Limitations of Multi-Analyte Approaches in Plant Biochemistry and Phytochemistry Research

After the central, but not new, let alone innovative, metabolomics research question “isn’t it advantageous to measure everything in order to capture all interrelationships?” had matured in the face of improved technological possibilities, the insight quickly came that access to scientific knowledge that goes beyond the mere description of the experiments conducted and data received is cumbersome [52]. This is probably also because in any “omics” (genomics, proteomics, metabolomics) research, the formulation of hypotheses is often either rather trivial (e.g., the measurable signal pattern of entity A differs significantly from the signal pattern of entity B) or is omitted a priori. If it is omitted, however, the researcher is either thrown back on the luck of serendipitous discovery or must reduce their science to the toil of inductive knowledge acquisition.

It still seems impossible for researchers to extract “meaningful” features or feature correlations and feature interdependences (e.g., network structures) from the accumulated data stream. The fundamental question of whether the chosen holistic approach of “omics” makes sense at all in a particular research setting is often not asked. On the contrary, it is assumed that if one axis (e.g., proteomics) is not sufficient, systems biology questions can only be adequately addressed by combining the individual stages of the mechanistic gene-phen cascade [53]. However, this question of meaning always arises, regardless of the chosen field of investigation, when a research approach tends to confuse or fail to separate irrelevant technical process noise from relevant biological process noise. [54]. This is more of a problem with inductive research approaches than with deductively guided experiments designed to test predefined questions, since phenotypic variability and plasticity can already be considered when formulating the research questions.

Over the past two decades, all sorts of technological efforts have been made to improve the interpretation (not necessarily the interpretability or classifiability) of experimental omics data. This has been done independently of questioning the meaning of the research itself and has been and is still driven by the assumption that the absence of usable results (e.g., “features” that represent possible biomarkers for the therapy and/or diagnosis of a disease) does not mean that such markers do not exist, but only that they have not been searched for well enough. This approach to data assessment allows further (in-depth) research to be defined as necessary and ethical whenever it seems convenient. [55,56].

In biological sciences, the bad habit of providing interpretations that are heavy with meaning for every research result has become widespread. In addition to publications that are intended to be addressed to the scientific community equipped with expert judgement, results that have been simplified to the point of distortion are regularly made accessible to the public as part of the individual or institutional marketing of activities without any further aid to reflection. If findings disseminated in this way are the results of inductive research, it is little wonder that factual knowledge exposed to falsification in deduction is replaced by belief in the sham knowledge made factual by populist publicity. In the best case, all swans remain white in the general perception; in the worst case, the “feature” that was observed first is elevated to incontrovertible fact. It is well known that popularized knowledge is difficult to eradicate. In the case of the dogma “all swans are white”, only the discovery of Australia with its black swans led to its falsification [57]. In the case of more complex topics, an epistemologically sound falsification of hypotheses that have once been accepted as facts in public discourse is very time-consuming and resource intensive.

The accelerated knowledge transfer on the brink of the current pandemic [58], which was largely decoupled from scientific peer review, has clearly shown that it is of extraordinary importance to structurally characterize discriminators (“statistically”) found in in silico datasets. This is independent of experimental data sources, such as relative copy numbers of specific mRNA polymers (genomics and expression patterns), the pattern differences in 2D protein gels or highly complex mass spectra attributable to the peptides of sample proteins (proteomics and peptidomics), or even the secondary metabolite patterns in biological samples (metabolomics). In these contexts, it is certainly not helpful to modify already complex research approaches into even more complex research approaches only because results such as “no significant differences were observable” are not satisfactory for the researcher. In contrast, inductive research approaches must be developed into deductive lines of evidence to be able to recognize formulated hypotheses as such in the first place. In this context, the structural characterization of the molecular entities (“molecules”) that represent discriminating features in the collected data provide a good starting point for further experiments [59,60]. These are necessary in those research cases where independent (deductive) experiments need to prove that the hypothesized discriminating features can be reproducibly recovered. In the currently cultivated mechanistic scientific worldview of actions and reactions, only the assignment of “features” to a molecular structure allows these distinguishing features to be rationally used for possible and desired diagnostic, therapeutic, or other scientific applications.

## 3. Fundamentals of NMR Spectroscopy

Since this review article deals with the use of NMR spectroscopy as a detection option for analytical chromatographic substance separations, the following paragraphs are intended to give the reader the opportunity to become familiar with the basics of this technology. For more detailed information, please refer to the literature [15,61,62].

### 3.1. Information Content of NMR Spectra

NMR spectra are information rich as each NMR active nucleus in an observed molecule (e.g., ^1^H, ^13^C—atoms) leads to an individual signal in that spectrum. The resonance frequency of the signal (“chemical shift”) and the signal structure (“coupling pattern”) are functions of the position of the observed atom in the molecular scaffold. Hence, NMR signal analysis allows reconstructing that scaffold. An extraordinary advantage of NMR spectroscopy over other spectroscopic methods and over mass spectrometry is that the area of an NMR signal (measured as a signal integral) is directly proportional to the number of atomic nuclei responsible for that signal. This means that, if for example a CH_3_-CH- structure element is present, the signal area of the methyl to methine group has a ratio of 3:1, provided easily realized experimental conditions, such as a sufficient signal-to-noise ratio, a good NMR signal shape, and a sufficiently high data density (digital resolution), are fulfilled. Since the comparability of the NMR signal areas is valid not only intramolecularly but also intermolecularly, relative (and if calibrated) absolute quantifications and hence quantitative NMR spectroscopy (qNMR) can be performed in substance mixtures [63]. However, it should be noted that deviations can occur if saturation effects occur due to excessively short relaxation times between individual experiments. Additionally, intensity deviations also occur when using decoupling techniques due to the nuclear Overhauser effect (NOE). Therefore, ^13^C-NMR spectra, which are usually recorded as decoupled single line spectra, cannot be evaluated quantitatively.

### 3.2. NMR Experiments

Due to principally different acquisition modes, NMR experiments can be classified by their dimensionality. In one-dimensional NMR spectroscopy (1D-NMR), NMR signals are recorded for a specific NMR active nucleus, in natural product research, usually ^1^H or ^13^C, rarely ^15^N or ^31^P. One-dimensional NMR spectra show NMR signals with all the details outlined above, but do not allow to deduce relationships between the nuclei directly from the spectrum. In two-dimensional NMR spectroscopy (2D-NMR), spin–spin correlations can be observed and made visible via plotting spin–spin correlations in a two-dimensional contour plot. If two dimensional NMR spectra are conducted such that correlation signals are detected between the atoms of identical isotopes (e.g., ^1^H-^1^H or ^13^C-^13^C), these are called homonuclear experiments. Correspondingly, if different isotopes are involved, these are heteronuclear experiments.

If NMR spectroscopy is hyphenated to separation techniques as liquid chromatography (HPLC-NMR), the NMR spectrometer is simply used as an on-line detection device of the chromatography effluent without changing the general technical setup of the spectrometer. The conventional sample container “NMR tube” is just replaced by the sample container “NMR flow cell”. Hence, all NMR experiments described for conventional NMR instruments are in principle available for online-NMR setups. However, due to experimental limitations as—to list the most important ones—the peak dilution in the chromatographic process, the limited mass capacity of analytical chromatography, the mismatch of chromatographic peak volume and NMR flow cell, the mismatch of peak duration, and NMR recording speed, only a limited number of NMR experiments are frequently used to tackle analytical problems in real-world settings.

#### 3.2.1. 1D-NMR Experiments

Due to the nuclear properties of the proton and due to its high relative abundance, the fastest NMR experiment is the observation of ^1^H nuclear resonances. It is therefore the basic experiment in the spectroscopic study of small molecules and mixtures of small molecules. Usually, information on the chemical shift and a rough estimate of the number of ^1^H nuclei in the molecule can be obtained, as well as first indications of the coupling partners of isolated signal groups. In addition, the absence or presence of structural elements (e.g., aromatic residues, methoxy groups, methyl groups without coupling partners, glycosides, etc.), can be of diagnostic value for the identification of the analyzed compound class. For example, several methyl groups without coupling partners probably indicate the presence of a mono-, di-, or triterpene, since the isoprene units that biogenetically give structure here have exactly these substituents. However, for a more detailed insight into the coupling network of the protons, at least homonuclear 2D shift correlation spectra must be recorded. If one wants to gain additional knowledge about the neighboring carbon centers (C-H pairs) correlating with the protons or to establish ^1^H-^13^C-^13^C coupling networks, heteronuclear 2D shift correlation spectra must be recorded [15].

#### 3.2.2. 2D-NMR Experiments

As discussed earlier, the incorporation of heteronuclear correlation experiments, which are based on scalar couplings, is at the heart of NMR-based structure elucidation [64]. Heteronuclear single-quantum (HSQC) or heteronuclear multi-quantum correlation (HMQC) experiments allow C-H bond pairs to be identified. Heteronuclear multiple-bond correlation (HMBC) experiments make it possible to link these building blocks into larger groups or to link C-H bond networks separated by heteroatoms (e.g., ether bridges or glycosidic bonds). However, it must be noted that HMBC correlations offer ambiguous solutions due to guidance over the selection of a particular coupling constant, which must be solved by combinatorial approaches [15,64]. Scalar neighborhood relations within the proton framework are elucidated by homonuclear double quantum filter correlation spectroscopy (DQF-COSY) and long-range total correlation spectroscopy (TOCSY) experiments. Due to the very limited signal capacity of the ^1^H NMR shift axis, signal overlap and ambiguities regarding the possible solutions often occur in these experiments. This is remedied by the combination of HSQC and TOCSY realized in the heteronuclear single quantum correlation–total correlation spectroscopy (HSQC-TOCSY) experiment. This enables the correlation of all protons within a spin system with the corresponding carbon atom framework.

If one wishes to study spatial neighborhood relations or verify spin systems derived from the combination of HSQC and HMBC experiments, it is recommended that homonuclear nuclear Overhauser effect spectroscopy (NOESY) or rotational nuclear Overhauser effect spectroscopy (ROESY) experiments are performed. With these experiments, neighboring protons are detected via dipolar coupling propagated through space. Due to the distance limitation of these experiments (usually 0.5 nm, equaling approximately five CH bond lengths), the correlation events of atoms localized in different proton spin systems indicate close spatial neighboring. In secondary natural products, this usually, but not necessarily, indicates a separation by no more than two-to-three non-protonated scaffold elements [15]. Both experiments are also valuable, if the relative configuration of an analyte needs to be investigated. However, the absolute configuration of an analyte cannot be deduced from NMR data which is an achiral physical method. Only if a chiral selector is introduced into the sample, e.g., by using a chiral solvent or by forming chiral complexes, can information about the absolute configuration of chiral carbon centers be deduced [15,64].

#### 3.2.3. Non-Uniform Sampling (NUS) in 2D-NMR Experiments

Non-uniform sampling (NUS) is an alternative recording method to conventional data acquisition for multi-dimensional NMR spectra. No complete data matrix is recorded; the stochastically guided omission of a part of the experiments saves the associated recording time. Reconstruction algorithms are used to reconstruct a correct spectrum from the patchy dataset; up to 75% of the matrix may be missing. Comparative experiments have shown that the resolution of spectra recorded with NUS is indistinguishable from conventional recordings. As a disadvantage, it must be noted that the sensitivity of NUS does not correspond to that of conventional recordings. In the case of quantity-limited samples, the tendency will therefore be to use the measurement time gained for more acquisition repetitions (scans) per matrix point. Alternatively, for heteronuclear spectra (HSQC, HMBC), the measurement time gained can be used to significantly increase the spectral resolution in the ^13^C-NMR axis [65,66]. Combining the NUS acquisition technique with the NOAH approach, wherein multiple 2D-NMR methods are concatenated in a single super-pulse sequence with a single relaxation delay for all spectra to be acquired, it is possible to further lower the required measurement time [67,68,69].

## 4. NMR Spectrometers as Detectors in Chromatography

Since organic molecules usually show very rich NMR spectra, it is advantageous to bring the substances under investigation in pure form for analysis. Therefore, the isolation of natural products is usually the prerequisite for their structural characterization. However, due to the metabolic complexity of the extracts obtained from natural products, this task is laborious and demanding. To overcome this limitation, the online combination of analyte separation by liquid chromatography and NMR spectroscopy (HPLC-NMR) was envisioned from the late 1970s onwards and realized in commercially available setups shortly thereafter [70]. From these pioneering works onwards, HPLC-NMR and related flow probe NMR technologies have evolved into well-established analytical platforms around the turn of the century [71,72,73].

### 4.1. HPLC-NMR

If one wants to convert a conventional NMR spectrometer, which is used with NMR tubes, into a detector designed to detect signals in a liquid flow, the measuring device itself and the NMR probe head, must be adapted to this measurement regime. Although conventional Helmholtz high-frequency coils (saddle coils) are used in HPLC-NMR flow probes, the dimensions must correspond to those of analytical HPLC in order not to lose sensitivity. While in conventional NMR spectrometers, an NMR sample tube is placed vertically in the center of these coils, in HPLC-NMR, this tube is replaced by a vertically mounted flow cell. This is connected to the HPLC module via a capillary, usually with the liquid flow entering the cell from the bottom and leaving it at the top. The mobile phase eluting from the HPLC column enters the flow cell in the HPLC-NMR and the NMR spectra can be recorded permanently. To improve the signal-to-noise ratio, multiple NMR experiments (e.g., 16 scans, with a recording time of less than one minute) are usually accumulated into one NMR spectrum.

**Figure 1:**
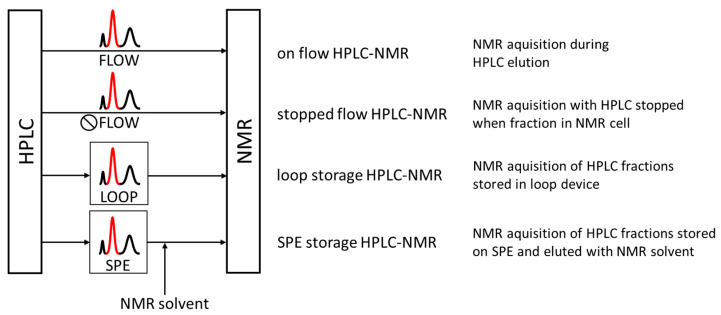
Illustration of the HPLC/NMR coupling modes. Direct coupling includes “on flow” and “stopped flow” setups, and indirect coupling makes use of the physical separation of chromatographic peaks in the mobile phase (“loop storage”) or on solid phases (“SPE storage”). Only in solid phase trapping, the HPLC mobile phase can be replaced with NMR solvent.

As with HPLC-MS, the acquisition time of these spectra must be adjusted to the average peak widths generated by the chromatographic system to allow the acquisition of one or more NMR spectra for a chromatographic peak. The main advantage of on-flow HPLC-NMR—namely online and real-time access to structure-rich NMR data from chromatographic peaks—was countered by several disadvantages. In addition to the inherently low sensitivity of NMR spectroscopy and the time limitation of the acquisition time due to the chromatographic peak width, it is the changing composition of the mobile phase with the common use of the HPLC solvent gradient that limited the applications of on-flow HPLC-NMR. Technological advancements in the coupling have therefore led to both the establishment of stopped-flow instrumentations and the temporal separation of chromatography and NMR using loop collection and loop storage peak parking devices (Figure 1) [70,71,72,73,74].

Whereas HPLC-NMR on flow experiments nearly vanished from the literature in recent years due to its insensitivity, the stopped flow and loop collection experiments are still being applied in natural product chemistry and the pharmaceutical industry, especially in combination with liquid handling robotics, even recently facilitating NMR multiplexing [75,76].

### 4.2. Capillary NMR

The introduction of capillary HPLC (capLC) for the analysis of mass limited samples made the development of alternative NMR probes for the capLC-NMR hyphenation necessary. The invention of capillary NMR (capNMR) probes with solenoidal RF coils led to the commercial availability of micro-coil probes with active sample volumes down to 1.5 µL [77,78]. Such probes either serve as detectors for flow-matched capLC equipment [79] or are combined with sample robots in industrial-style high-throughput setups [80].

**Figure 2:**
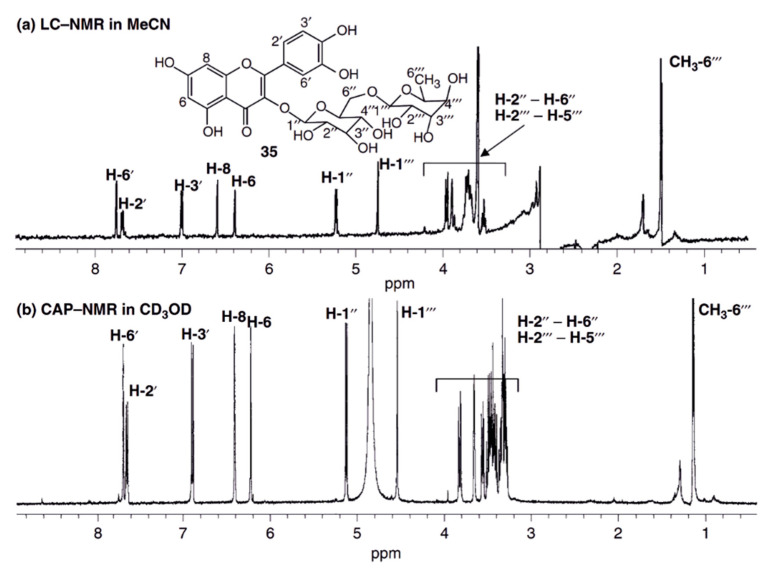
Comparison of the 1H NMR spectra of 20 µg rutin recorded by the stopped flow HPLC-NMR (**a**) and by capNMR (**b**). Both spectra were recorded on a 500 MHz spectrometer. Due to the higher sensitivity of the capNMR probe-head, the acquisition of the capNMR spectrum took only 10% of the HPLC-NMR measurement time. The S/N ratio of the capNMR spectrum is better than in HPLC-NMR and utilizing a deuterated NMR solvent (methanol-d_4_), lead to an undisturbed baseline since no experimental suppression of the solvent signal had to be performed. Reprint from Wolfender et al. [73] with permission from Wiley.

Alternatively, capNMR was used offline with samples taken up in a few microliters of NMR solvent and manually introduced into the probe or by the aid of a syringe pump [81]. Whenever an analyte was not concentration-limited in the NMR solvent due to poor solubility, micro-coil NMR was found to be superior to conventional NMR machines due to the inherently higher sensitivity of smaller-diameter coils (Figure 2). The up to five-fold sensitivity gain of cap NMR setups made it possible to record heteronuclear 2D-NMR spectra for sub-milligram analyte quantities not analyzable by conventional NMR instruments [82].

### 4.3. HPLC-SPE-NMR

**Figure 3:**
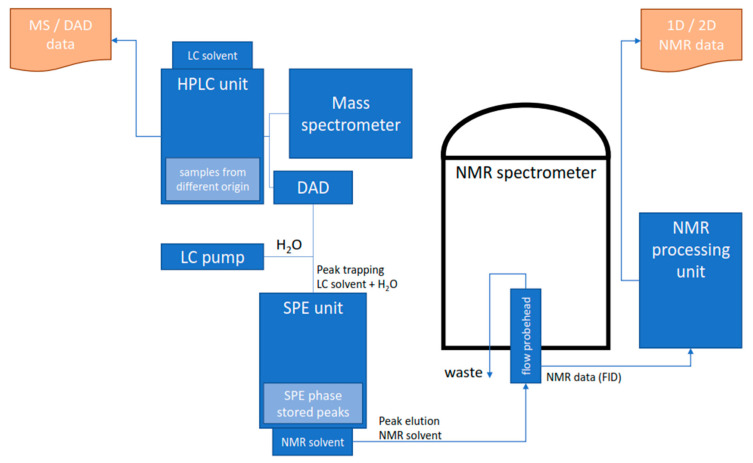
Schematic diagram of an HPLC-MS/DAD-SPE-NMR system. Analytes separated in the HPLC system are trapped post-column in an SPE device using a make-up flow to increase the effluent polarity. Trapping events are triggered by UV or MS signals; hence, automation and multiple trapping events are possible. The SPE cartridges are rinsed and dried to remove the HPLC solvent; and the subsequent analyte transfer to the NMR spectrometer is performed with an appropriate NMR solvent, e.g., ACN-d3, MeOD-d4, or CDCl_3_.

When Griffiths and Horton described an analytical setup approximately 25 years ago, in which, after the analytical separation of analytes in HPLC, they were enriched on another lipophilic stationary phase to be transferred to an NMR solvent, the concept of high-performance liquid chromatography solid phase extraction nuclear magnetic resonance (HPLC-SPE-NMR) was invented [83]. Their setup allowed to isolate individual peaks, remove the mobile phase of the HPLC by washing the stationary phase with a solvent of low eluotropic strength, and transfer the isolated analytes to the flow-through head of the NMR spectrometer using suitably eluotropic NMR solvents (e.g., CD_3_OD, CD_3_CN) (Figure 3). They found that the achievable signal intensities were inversely proportional to the analytical chromatographic peak volumes, leading to the conclusion that an elution volume close to the flow cell volume of the HPLC-NMR probe head leads to an optimal sensitivity improvement. This workflow and the argument to match HPLC peak volumes with the NMR flow cell volumes are major keystones for the successful application of HPLC-SPE-NMR [10,16,72]. A highly automated and refined HPLC-SPE-NMR setup allowing full control over HPLC-UV or HPLC-MS triggered trapping events, solid phase extraction (SPE) cartridge handling in a 96-well plate format, and analyte elution from the SPE to either an NMR spectrometer or another collecting device (auto sampler, NMR tubes, etc.) is available from a major NMR manufacturer [84].

In typical natural product analysis setups, the sensitivity of HPLC-SPE-NMR is more or less comparable to capNMR. As a rule of thumb for a typical secondary metabolite with a molecular weight of ~500 Da, approximately 2 μg analyte/μL NMR solvent must be transferred to the active HPLC-SPE-NMR probe volume (e.g., 120 μg analyte into a 60 μL flow probe with a 30 μL active cell volume mounted in a 600 MHz NMR spectrometer equipped with a room temperature probe-head) to obtain homo- and heteronuclear 2D-NMR spectra (e.g., DQF-COSY, TOCSY, HSQC, and HMBC) overnight [10]. If only ^1^H-NMR spectra are required (e.g., for metabolic fingerprinting purposes), approximately 10 nmol substance (5 μg at 500 Da) are needed to obtain sufficiently good NMR spectra within one hour of measurement time [85,86].

The advantages of HPLC-SPE-NMR over HPLC-NMR are the avoidance of H_2_O signals in NMR spectroscopy, which have necessitated signal suppression, the significant cost savings because deuterated NMR solvents no longer need to be used as components of the mobile phases of HPLC, and the independence of NMR spectrum acquisition from the eluents of gradient HPLC. It must be mentioned, however, that the careful optimization of experimental conditions is usually required to ensure the optimal transfer of analytes from the HPLC domain to the NMR spectrometer [87]. Special attention must be paid to the interaction of the analytes with the stationary phase of the “trap column”. Since this is usually a conventional material, such as divinylbenzene type polymer or RP-C18 silica, which is well known from solid phase extraction (SPE), the experimental rules of off-line SPE can be applied here. For example, it is possible to load the SPE columns multiple times, which leads to a massive increase in the analyte concentration in the NMR probe. At the same time, however, it must not be overlooked that post-HPLC, peak enrichment by means of lipophilic phases means a significant limitation of the analytical scope. Analytes that cannot be precipitated at the SPE stationary phase are lost for subsequent transfer to the NMR spectrometer. In such cases, alternative approaches such as capNMR must be pursued [88].

## 5. Applications Examples

### 5.1. HPLC-NMR

Phytochemical application examples for the early adoption of on-flow, stopped-flow, and loop-collection/loop-storage HPLC-NMR setups (Figure 1) include successful secondary metabolite identification from a multitude of plants such as *Ancistrocladus griffithii* [89], *Potamogeton lucens* [90], *Drosera* ssp. [91], over a wide variety of analyte classes, as phytosterols [92], polyphenols [93,94], xanthons [95], or flavonoids [96] and included in vitro bio-transformation [97] and bio-degradation studies [98].

### 5.2. Capillary NMR

This setup was successfully used in an offline mode to characterize the mass-limited natural product samples within decent NMR acquisition times, e.g., an overnight recording of a HSQC and a HMBC spectrum for 100 µg of a ~500 Da analyte (~40 mM solution) [82,99,100]. If the manual sample delivery used in some multi-sample capNMR approaches [101,102] is replaced by automated liquid handling devices, large sample arrays can be handled in the sophisticated “microdroplet” [103] or “segmented flow analysis” NMR [104,105]. In recent decades, this idea was further developed into “multiplex NMR” with several capillaries mounted in one spectrometer [76]. By hyphenating capNMR probes to capLC devices, true “LC-cap-NMR” hyphenations were realized. In this realm, the online structural characterization of mass limited samples, parallelization, miniaturization, and hyphenation to electrophoretic separation devices or gas chromatography were in the research focus [106,107,108,109].

### 5.3. HPLC-SPE-NMR

As of today, HPLC-SPE-NMR is the best established HPLC/NMR hyphenation technology. If published data are taken as a measure, natural product analysis is the major application field for this technology, but it must not be overlooked that any (as stated further above) chemical investigation with the need to structurally characterize an organic molecule will strongly rely on NMR technologies—including HPLC/NMR hyphenations.

In natural product analysis, two major HPLC-SPE-NMR sample preparation strategies have emerged [12]. If working with extracts which have undergone only little pre-analytical purification steps, multiple peak trapping is to be applied. Frequently, up to ten chromatographic runs are repeated and a dozen or more analytes are trapped. Since the runtimes of the LC assays of crude extracts usually range between 40 and 90 min, such an operation is usually performed overnight and uses up to 500 µL of the LC sample. The subsequent transfer of the isolated peaks to the NMR spectrometer and recording of its NMR spectra is performed peak by peak. Another possible strategy starts with a comprehensive pre-analytical sample preparation protocol tailored to obtain a set of fractions enriched with a small subset of the questioned metabolites. Hence, a higher fractional amount of the injected sample is the desired metabolite, and the number of trapping events can be reduced to one. An advantage of such an approach lies in the fact that analytes do not have to be stored on the SPE device for long.

A multitude of publications describe its use to dereplicate the plant extracts of different origin, e.g., from *Corydalis solida* tubers [110], from plants with traditional medicinal use such as *Harpagophytum procumbens* (Devils Claw) [86], *Hypericum perforatum* (St. John’s Wort) [111], *Morus alba* [112], or from plants with uncharted bioactivity profiles such as *Carthamus oxyacantha* [113] and startling novel fungal compound classes as chiral dispiro-derivatives [114]. Unraveling the secondary metabolite profile in the model plant *Medicago truncatula* was facilitated with the aid of this hyphenation [115] as it was previously described for tomato metabolite profiling [116]. HPLC-SPE-NMR orientated research is always oriented towards the structural characterization of analytes. Major metabolite classes addressed in recent years were, for example, alkaloids [117,118,119,120,121], diarylheptanoids [122], flavonoids [123,124], isoflavonoids [125], chromenes [126], lignans [127,128], phenanthrenes [129], terpenoids of different sub-classes [130,131,132], steroids [133,134], iridoids [135], polyphenols [136], and saponins [137,138].

HPLC-NMR [139] and HPLC-SPE-NMR [140] were utilized to elucidate the absolute configuration of natural products by the Mosher ester derivatives’ measurement of phloroglucinol congeners and polyacetylene derivatives, respectively. In a recent vitetrifolin D metabolization study with liver cell microsome incubation experiments HPLC-SPE-NMR was successfully utilized to structurally characterize nine phase I metabolites only tentatively identified by high-resolution HPLC-MS approaches [141].

**Figure 4:**
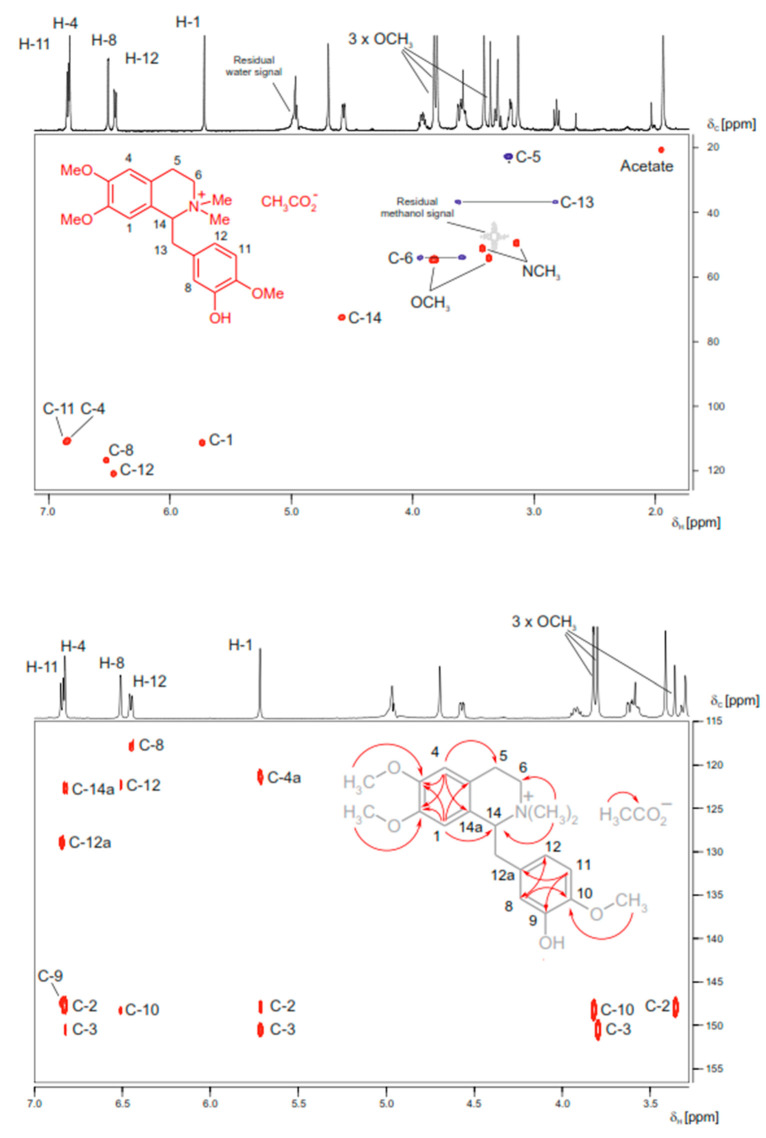
HPLC-SPE-NMR-derived HSQC (top figure) and HMBC (bottom, figure) spectra of *N*-methyllaudanidinium acetate (structure see formula insert); a secondary metabolite found in *Corydalis solida* tubers. Online structure elucidation was facilitated from an aliquot of an alkaloid-enriched solid phase extraction fraction (2.1 mg) obtained from the methanolic extract (93 mg) of a single 233 mg *Corydalis solida* tuber. The NMR spectra were obtained in deuterated methanol on a 600 MHz NMR spectrometer, equipped with a 30 μL cryofit insert fed into a 3 mm DUI cryoprobe. HSQC (6-hour acquisition time): Red cross-peaks are stemming from CH and CH_3_ protons, blue cross peaks from CH_2_ protons. Residual solvent signals are given in grey. HMBC (12-hour acquisition time): The correlation information derived from the marked cross-peaks is summarized in the formula insert. Adapted from Sturm et al. [110] with permission from Elsevier.

As in conventional NMR spectroscopy, in HPLC-SPE-NMR applications, the number of NMR spectra acquired depends on the complexity of the structural elucidation problem. If the molecular formula and compound class are known (e.g., from HPLC-MS experiments and from biogenetic considerations), the metabolites of intermediate complexity, such as flavonoids, alkaloids, or iridoid glycosides, can be identified by easily accessible 1H NMR spectra, if necessary. However, if highly complex 1H spin systems are encountered or if the species under study has not yet been well phytochemically described, a “complete spectra set” of homo- and heteronuclear 2D-NMR spectra must be acquired to obtain ^13^C NMR shift information [142,143]. Here, it is of great importance to maximize the amount of substance in the flow cell of the NMR probe head and to increase the sensitivity of the spectrometer (e.g., by using ultra-cooled electronics) to keep the experiment times low (Figure 4).

If for instrumental setup reasons (for example, if an available high-end NMR spectrometer from one vendor was not compatible to the HPLC-SPE front-end from another vendor), HPLC-SPE-NMR is not possible, then alternative approaches can be pursued. For example, the HPLC-SPE instrument can be operated as an independent analytical unit. The effluent from the SPE column (analyte in NMR solvent) is not fed online via capillary to the NMR spectrometer but is collected in alternative collection vessels. The working group around Jerzy W. Jaroszewski, who died much too early in 2011, was able to show in 2007 that samples purified and isolated by HPLC-SPE in Copenhagen could be transported to Geneva without any problems to be measured on a capNMR measuring device. Due to the filtering effect of the SPE column, similar concentration profiles were obtained as with HPLC-SPE-NMR; the sample was free from interfering additions of the mobile phase or impurities from the sample containers [144]. In an alternative approach, SPE trapped analytes can be transferred to conventional or low-volume NMR sample tubes. Again, the SPE serves as a purification step and the preparative workup of an extract to obtain the analyte of interest is avoided [145,146,147,148,149,150].

In summary, NMR spectroscopy can be used very successfully as a detector in high-performance liquid chromatography. The instrumentation, which is currently only available from one manufacturer, appears to be mature—in both HPLC-NMR and HPLC-SPE-NMR. Additionally, the exploration of NMR spectroscopic characterization of minute amounts of substances has led to advances in the development of micro-technologies. Finally, it must be positively noted that due to the use of conventional spectrometer technology in HPLC-NMR coupling, any progress in NMR method development can also benefit the application in coupling.

## 6. Conclusions

The aim of this review was to show the reader that it is necessary to strengthen holistic research approaches (“omics”) with verification strategies—in particular, when it is necessary or desirable to statistically subject discriminators in an analytical dataset to verification. If chromatography with mass spectrometric detectors is used in “omics”, it is usually impossible to carry out this verification in such a way that a discriminator (“putative bio-marker”) can be unambiguously assigned, i.e., structurally proving, to a chemical compound.

The authors strongly recommend the use of NMR spectroscopy to support the elucidation of the molecular structures of target molecules or—in the best case—to make this possible. Sustainable mass spectrometry-based metabolomics research must have access to facilities that are able to perform the structural elucidation of organic molecules by NMR. If this is not possible, the identification of possible biomarkers is usually reduced to known reference substances or too often dubious assignments via databases, which per se also only contain secondary substances that are already well known. Coupling NMR spectrometers with chromatographic equipment, optimally by using the HPLC-SPE-NMR hyphenation, not only facilitates the assignment of discriminatory features of LC-MS to NMR signals, but also avoids or at least reduces the extremely resource-intensive work of isolating target molecules.

The scientific literature impressively shows that wherever an instrumental installation is available to combine analytical chromatography with NMR spectroscopy, it is also used to successfully characterize unknown substances in biological materials. The scientific community impressively and continuously demonstrates that NMR spectroscopy is one of the conventional structure-elucidating technologies and that mass spectrometric/spectroscopic data are usually not sufficient to characterize organic compounds with sufficient confidence.

The authors would like to emphasize that it is their opinion that the analytical industry is currently not sufficiently supportive of this approach. After the loss of a major NMR manufacturer due to takeover by a major manufacturer of mass spectrometers and chromatographic equipment with the discontinuation of business about a decade ago, there are only two manufacturers of high-field NMR spectrometers left in the scientific world. Only one of them is active in the field of coupling chromatography and NMR spectroscopy. Technological progress in this field is therefore extremely limited; no significant technological breakthroughs or advancements have been observed in recent years.

It is to be feared that the coupling of chromatography with NMR spectroscopy has reached its zenith and that the industry will abandon this branch of technology. Thus, two to three decades in technological development will soon be lost and it will be the task of the following generations of researchers to realize that the conquest of metabolic diversity in metabolomics and metabonomics is pointless if the encountered signal diversity cannot be converted into structural knowledge, into new molecules, and thus new biomarkers.
